# The COVID-19 Pandemic Was Associated with a Change in Therapeutic Management and Mortality in Heart Failure Patients

**DOI:** 10.3390/jcm13092625

**Published:** 2024-04-29

**Authors:** Matteo Ponzoni, Gabriella Morabito, Giovanni Corrao, Gino Gerosa, Anna Cantarutti, Federico Rea

**Affiliations:** 1 Cardiac Surgery Unit, Department of Cardiac, Thoracic, Vascular Sciences, and Public Health, University of Padua, 35128 Padua, Italy; gino.gerosa@unipd.it; 2 National Centre for Healthcare Research and Pharmacoepidemiology, 20126 Milan, Italy; gabriella.morabito@unimib.it (G.M.); giovanni.corrao@unimib.it (G.C.); anna.cantarutti@unimib.it (A.C.); federico.rea@unimib.it (F.R.); 3 Department of Statistics and Quantitative Methods, University of Milano-Bicocca, 20126 Milan, Italy

**Keywords:** heart failure, COVID-19, heart transplant, mechanical support device

## Abstract

*Background:* Heart failure (HF)-related mortality has been exacerbated by the COVID-19 pandemic; however, it is unclear how healthcare reassessment has contributed to the excess mortality versus SARS-CoV-2 infection itself. We aimed to assess how the pandemic affected the therapeutic management and prognosis of HF patients. *Methods:* We retrospectively reviewed the healthcare utilization databases of the Lombardy region (Italy) to identify all newly-diagnosed HF patients from January 2018 to December 2021. Outcomes were the utilization of HF therapies (Sacubitril/Valsartan; cardiac resynchronization therapy [CRT] and/or implantable cardioverter-defibrillator [ICD]; mechanical circulatory support [MCS]; heart transplantation [HTX]) and mortality. Cox regression models were fitted to estimate the hazard ratios (HR) and 95% confidence intervals (CI) for outcomes associated with the pandemic. *Results:* 36,130 and 17,263 patients were identified in the pre-pandemic and pandemic eras, respectively. The pandemic reduced Sacubitril/Valsartan utilization (HR = 0.77, 95% CI: 0.65–0.91) and CRT/ICD implantation (HR = 0.85, 95% CI: 0.78–0.92), but not MCS (HR = 1.11, 95% CI: 0.86–1.43) and HTX (HR = 0.88, 95% CI: 0.70–1.09). An increased mortality risk was observed during the pandemic (HR = 1.19, 95% CI: 1.15–1.23), which was attributable to SARS-CoV-2 infection (HR for non-COVID-19-related mortality = 1.01, 95% CI: 0.97–1.04). *Conclusions:* The COVID-19 pandemic was associated with a reduction in medical and interventional therapies for HF and an increase in mortality for HF patients.

## 1. Introduction

The COVID-19 pandemic has recently been associated with a significant increase in heart failure (HF)-related mortality in a large US nationwide data analysis [1]. It is known that SARS-CoV-2 infection can exacerbate myocardial injury in patients with chronic HF by generating a high inflammatory status, endotelitis, and myocarditis-like cardiac damage [2]. Severe COVID-19 can trigger a pro-coagulative state, characterized by high D-dimer, thrombin, von Willebrand factor, and lupus anticoagulant levels, leading to a higher risk of thrombotic events, a need for mechanical ventilation, and adverse outcomes [3]. Moreover, the cytokine storm in response to SARS-CoV-2 infection can increase platelet production and function, which can predispose to disseminated microthrombosis in different organs and diffuse myocardial injury [3].

In parallel, the COVID-19 pandemic generated the need for an important reassessment of healthcare resources and policies, with significant consequences in limiting and/or delaying the patient’s access to hospital care [4,5,6,7]. In particular, a decreasing trend in HF hospitalizations during the first waves of the COVID-19 pandemic was reported, together with changes in interventional and surgical practice [4,8]. The STS-INTERMACS registry documented a 17% and 23.5% decline in durable LVAD implantations in 2020 and 2021, respectively, compared to 2019 [9]. Conversely, heart transplantation (HTX) trends in the pre-pandemic and pandemic eras varied significantly across centers [10,11,12,13]. While some institutions reported unchanged rates of HTX procedures across the different pandemic waves, other large multicenter registries observed a deflection in HTX activity, especially in extreme ages [10,11,12,13]. However, the role of modifying patients’ access to HF care in exacerbating HF-related excess mortality during the COVID-19 pandemic is unclear.

In the present study, we aimed to assess how the COVID-19 pandemic affected the therapeutic management of HF in the Lombardy Region using a large multicenter database and evaluate the impact of the pandemic on the prognosis of patients with newly-diagnosed HF. In particular, we explored how the utilization of index therapies for HF (medical, interventional, and surgical) and the survival of HF patients changed from the pre-pandemic era to the pandemic era.

## 2. Materials and Methods

We conducted a retrospective observational study using the healthcare utilization databases of all residents of Lombardy, an Italian region that accounts for almost 16% of national population (~10 million residents), which is entirely covered by the National Health Service (NHS). These databases provide precise information on all reimbursable health services such as outpatient drug prescriptions, inpatient and outpatient surgical interventions, primary and secondary diagnoses of hospitalizations, and death and are linked by a single individual identification code, which allows one to trace the healthcare pathway of NHS beneficiaries. The study was conducted according to the Declaration of Helsinki. Given the retrospective nature of the study, without direct contact with patients, approval from an ethics review board is not needed (according to the rules from the Italian Medicines Agency, available at: http://www.agenziafarmaco.gov.it/sites/default/files/det_20marzo2008.pdf; accessed on 30 January 2024).

All Lombardy residents aged ≥18 years and with the first hospital diagnosis of HF from January 2018 to December 2021 were identified and followed up from the date of the first hospitalization until death, emigration, or 31 December 2021. Patients were classified into a pre-pandemic or pandemic group, i.e., diagnosed before and after 1 March 2020, respectively. High-dimensional propensity score (HDPS) [14] was applied to account for possible differences between groups. Briefly, the propensity to be diagnosed during the pandemic was estimated through a logistic regression model, which included the 200 most predictive covariates among all drugs dispensed and hospital diagnoses experienced in the three years before the HF hospitalization.

Primary outcomes were the utilization of index HF therapies: prescription of Sacubitril/Valsartan (medical management); implantation of cardiac resynchronization therapy (CRT) and/or implantable cardioverter-defibrillator (ICD) (interventional management); mechanical circulatory support (MCS) and heart transplantation (HTX) (surgical management). 

Cox regression models were fitted to estimate the hazard ratios (HR) and 95% confidence intervals (CI) for primary outcomes associated with the COVID-19 pandemic, included as a time-dependent variable. Models were adjusted for several demographic and clinical covariates (see Table 1) and HDPS.

To evaluate the differences in mortality rates between the two time periods, two analyses were performed: (i) Cox regression models and (ii) cumulative incidence function (CIF). Among patients enrolled during the COVID-19 pandemic, overall mortality was depurated from SARS-CoV-2 mortality, defined as at least a sign of infection in the month preceding death. Thus, two Cox regression models were fitted to estimate the HR and 95% CI of, respectively, overall mortality and non-COVID-19-related mortality (COVID-19-related mortality was considered as a competing event for this latter). To calculate CIF, patients enrolled during the pandemic were matched for HDPS to patients enrolled in the pre-pandemic period, whose follow-up was censored to 1 March 2020. Cox models were adjusted for all the covariates, including HDPS.

## 3. Results

In the pre-pandemic and pandemic era, 36,130 and 17,263 patients, respectively, with newly-diagnosed HF were identified. Demographic characteristics and the burden of concomitant medications and comorbidities are summarized in Table 1.

Sacubitril/Valsartan was prescribed to 3.5% and 3.8% of patients before and during the pandemic, respectively. CRT/ICD was implanted in 4.1% and 2.7% of patients, MCS was used in 0.4% and 0.3%, and HTX was performed in 0.6% and 0.3% in the pre-pandemic and pandemic eras, respectively (Table 2).

According to the Cox model, the COVID-19 pandemic was associated with a significant reduction in the utilization of Sacubitril/Valsartan (HR = 0.77, 95% CI: 0.65–0.91, model adjusted also for calendar time, given that Sacubitril/Valsartan prescriptions showed a clear trend during the study period) and implantations of CRT/ICD (HR = 0.85, 95% CI: 0.78–0.92), but it had no significant effect on the probability of receiving an MCS (HR = 1.11, 95% CI: 0.86–1.43) or undergoing a HTX (HR = 0.88, 95% CI: 0.70–1.09).

**Figure 1:**
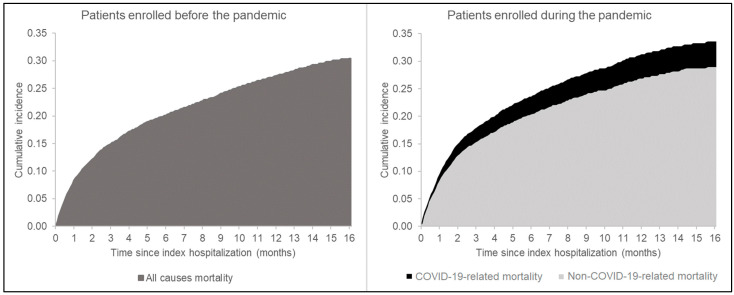
Cumulative incidence of mortality in the pre-pandemic and pandemic eras, stratified in COVID-19-related and non-COVID-19-related mortality.

The COVID-19 pandemic was associated with a 19% increased mortality risk (95% CI: 15%–23%). A total of 11,954 individuals from the pandemic era were then matched with as many patients enrolled before the pandemic. The overall cumulative incidence of death was higher in the pandemic matched-cohort compared with the pre-pandemic one (Figure 1). An overall 4% excess mortality during the pandemic era (at 12 months) was attributable to SARS-CoV2 infection. Patients implanted with MCS displayed a higher cumulative incidence of death, especially during the pandemic era (40% vs. 34% in the pandemic and pre-pandemic era at 12 months, respectively, *p* = 0.307) (Table 2). According to the Cox model, non-COVID-19-related mortality risk during the pandemic was similar to the pre-pandemic mortality risk (HR = 1.01, 95% CI: 0.97–1.04).

## 4. Discussion

Our work highlights the changes in HF management across the COVID-19 pandemic eras for newly-diagnosed HF patients. Using a large electronic health record database covering a population of ~10 million residents, we identified a significant impact of the COVID-19 pandemic in reducing the utilization of medical (−23%) and interventional therapeutic strategies (−15%) for HF. To assess these modifications, we analyzed the changes in the prescription of index medications for advanced HF (Sacubitril/Valsartan), as well as the implantation of CRT/ICD, MCS, and HTX procedures.

Our data align with the lower access of patients to health system resources [6,7,15] and electrophysiology procedures [8] recorded during the pandemic. Boriani and colleagues observed a 40% reduction of elective pacemaker, ICD, and CRT implantations during the first pandemic wave (compared to 2019), with a return to pre-pandemic activity intensity only during the third and fourth waves [8]. In a similar real-world healthcare utilization-based study in Germany, Kerwagen et al. documented a significant decrease in the quarterly growth rate of Sacubitril/Valsartan prescriptions in concomitance with the COVID-19 pandemic [16]. Interestingly, the treatment initiation remained compromised for 1 year [16], revealing a potential persistent therapeutic inertia, which mirrors our present findings. 

Conversely, our analysis revealed that surgical strategies for end-stage HF (namely short- and long-term MCS and HTX) were not significantly influenced by the pandemic. These data suggest an acceptable preservation of the referral and treatment of most severe HF cases in Lombardy during the pandemic. In fact, important updates and adjustments in HT donor selection, as well as HT recipient monitoring and surveillance, were introduced in Italy at the beginning of the COVID-19 pandemic [12]. Similarly, ad hoc novel guidelines for cardiac surgical patient screening at admission and during hospitalization, isolation measures, and intraoperative safety measures for patients and healthcare personnel were produced by the Italian Society of Cardiac Surgery in response to the COVID-19 outbreak [6,7]. However, the under-treatment of less severe patients might have led to an increase in candidates for advanced therapies, possibly mitigating the difference between the two eras.

As previously reported [1], our analysis confirms an increased (+19%, *p* < 0.001) excess risk of mortality for HF patients during the COVID-19 pandemic (Figure 1). These data parallel the results of Zuin and colleagues, who observed an increase in HF-related mortality in 2020 of 13.2% and 25.9% compared to 2019 and 2018, respectively [1]. In our work, after HDPS matching, the pandemic era was confirmed to be associated with a higher overall cumulative incidence of death in the HF population compared to the pre-pandemic period (31% vs. 27% at 12 months, respectively, *p* < 0.001). Interestingly, the 4% excess mortality during the pandemic was attributable to SARS-CoV2 infection, while non-COVID-19-related mortality risk in the pandemic era was similar to the pre-pandemic mortality risk. Although the identification of COVID-19 related mortality events presents inner limitations, our data support the deleterious effects of SARS-CoV2 infection itself in the fragile population of patients suffering from HF [2,3]. Additional investigations are necessary to better extrapolate the very direct and long-term consequences of the healthcare system reassessment on the prognosis of HF patients.

## 5. Limitations

Our work presents several limitations. The analysis of healthcare databases allows proper tracking of the utilization of healthcare resources (medications, interventions, surgeries, and hospitalizations) by a large number of patients over a long period of time. However, our databases lack additional clinical variables, such as the New York Heart Association functional class, the echocardiographic metrics of ventricular function, and other less frequent comorbidities, which may act as confounding variables. Although adjusting for HDPS should minimize potential biases, the severity of the disease may differ between patients admitted before and during the pandemic, and results may suffer from residual confounding. Therefore, we cannot make any argumentations about the relationship between the observed (slight) therapeutic management modifications and the increased risk of mortality during the pandemic. Moreover, we were not able to analyze the causes of death in our databases, which may give additional information to clarify the relationship between SARS-CoV-2 infection and the observed excess mortality in HF patients. Future studies should address also the quality of healthcare delivery during pandemics, as well as the patient’s perception and reaction to changes in healthcare policies, to further understand how healthcare reorganization during pandemic periods can actually impact the prognosis of patients.

## 6. Conclusions

Our work further corroborates (i) the indirect consequences of the COVID-19 pandemic on the reorganization of health care systems, organ availability, and the diagnostic-therapeutic management of HF and (ii) the detrimental association of SARS-CoV2 infection/inflammatory status and pre-existent cardiovascular diseases leading to HF, whose understanding is mandatory to implement pandemic preparedness and preventive strategies in extremely delicate populations. Further longitudinal studies are awaited to determine the very long-term effects of the COVID-19 pandemic on HF management strategies and their impact on patient outcomes. Moreover, the quality of healthcare delivery as perceived by patients and healthcare providers should be explored to optimize hospital policies during pandemic emergencies.

## Figures and Tables

**Table 1:** Demographic characteristics of the cohort. Abbreviations: SD: standard deviation; RAS: renin-angiotensin-system; MRA: mineralocorticoid receptor antagonist. * Multisource Comorbidity Score is a comorbidity index obtained from inpatient diagnostic information and outpatient drug prescriptions, validated using Italian data. The standardized mean differences were used to compare differences between groups. Standardized mean differences <0.10 were considered negligible.

	Patients Enrolled before the Pandemic (N = 36,130)	Patients Enrolled during the Pandemic (N = 17,263)	Standardized Mean Differences
Men	17,666 (48.9%)	8677 (50.3%)	0.018
Age (years), mean [SD]	80.3 [11.2]	80.1 [11.2]	0.037
RAS inhibitors/beta-blockers/MRA	44,883 (83.9%)	17,688 (82.6%)	0.033
Previous hospitalizations			
Stroke	1566 (4.3%)	709 (4.1%)	0.011
Myocardial infarction	1495 (4.1%)	679 (3.9%)	0.010
Cancer	2921 (8.1%)	1319 (7.6%)	0.017
Diabetes	2549 (7.1%)	1127 (6.5%)	0.021
Respiratory disease	5349 (14.8%)	2511 (14.6%)	0.007
Kidney disease	2533 (7.0%)	1047 (6.1%)	0.038
Number of medical co-treatments			0.026
0–4	11.336 (31.4%)	5582 (32.3%)	
5–9	15,291 (42.3%)	7290 (42.2%)	
≥10	9503 (26.3%)	4391 (25.4%)	
Multisource Comorbidity Score *			0.176
0 ≤ score ≤ 4	10,246 (28.4%)	5126 (29.7%)	
5 ≤ score ≤ 9	11,862 (32.8%)	5907 (34.2%)	
10 ≤ score ≤ 14	7326 (20.3%)	3325 (19.3%)	
15 ≤ score ≤ 19	34,943 (9.7%)	1514 (8.8%)	
Score ≥ 20	3202 (8.9%)	1391 (8.1%)	

**Table 2:** Frequencies of the treatments and cumulative incidence of mortality in the pre-pandemic and pandemic eras. CRT: cardiac resynchronization therapy; HTX: heart transplantation; ICD: implantable cardioverter-defibrillator; MCS: mechanical circulatory support.

Treatment Frequencies		Patients Treated before the Pandemic	Patients Treated during the Pandemic
Sacubitril/Valsartan		1855 (3.5%)	2047 (3.8%)
CRT/ICD		2170 (4.1%)	1419 (2.7%)
MCS		196 (0.4%)	144 (0.3%)
HTX		301 (0.6%)	171 (0.3%)
Cumulative Mortality	Time (months)	Patients enrolled before the pandemic	Patients enrolled during the pandemic
			Overall
Overall	6	0.20	0.24
	12	0.27	0.31
	16	0.30	0.34
Sacubitril/Valsartan	6	0.04	0.02
	12	0.07	0.05
	16	0.08	0.06
CRT/ICD	6	0.03	0.05
	12	0.06	0.09
	16	0.09	0.10
MCS	6	0.31	0.38
	12	0.34	0.40
	16	0.34	0.41
HTX	6	0.07	0.06
	12	0.07	0.09
	16	0.09	0.10

## Data Availability

The data that support the findings of this study are available from the Lombardy Region, but restrictions apply to the availability of these data, which were used under license for the current study and so are not publicly available. Data are, however, available from the corresponding author upon reasonable request and with permission of the Lombardy Region.
